# AcImpute: a constraint-enhancing smooth-based approach for imputing single-cell RNA sequencing data

**DOI:** 10.1093/bioinformatics/btae711

**Published:** 2025-03-03

**Authors:** Wei Zhang, Tiantian Liu, Han Zhang, Yuanyuan Li

**Affiliations:** School of Mathematics and Physics, Wuhan Institute of Technology, Wuhan 430205, China; School of Mathematics and Physics, Wuhan Institute of Technology, Wuhan 430205, China; School of Mathematics and Physics, Wuhan Institute of Technology, Wuhan 430205, China; School of Mathematics and Physics, Wuhan Institute of Technology, Wuhan 430205, China

## Abstract

**Motivation:**

Single-cell RNA sequencing (scRNA-seq) provides a powerful tool for studying cellular heterogeneity and complexity. However, dropout events in single-cell RNA-seq data severely hinder the effectiveness and accuracy of downstream analysis. Therefore, data preprocessing with imputation methods is crucial to scRNA-seq analysis.

**Results:**

To address the issue of oversmoothing in smoothing-based imputation methods, the presented AcImpute, an unsupervised method that enhances imputation accuracy by constraining the smoothing weights among cells for genes with different expression levels. Compared with nine other imputation methods in cluster analysis and trajectory inference, the experimental results can demonstrate that AcImpute effectively restores gene expression, preserves inter-cell variability, preventing oversmoothing and improving clustering and trajectory inference performance.

**Availability and implementation:**

The code is available at https://github.com/Liutto/AcImpute.

## 1 Introduction

scRNA-seq methods can enable the high-throughput and high-resolution transcriptomic analysis of individual cells, providing an additional dimension to transcriptomic information compared to traditional bulk sequencing (Kolodziejczyk *et al.* 2015). This technology has become the most advanced method to reveal the heterogeneity and complexity of RNA transcripts within individual cells (Cao *et al.* 2017, Jovic *et al.* 2022). Several sequencing protocols have been proposed for single-cell RNA sequencing. However, a prevalent challenge in single-cell sequencing data is that a very high percentage of genes in a cell are expressed with zero values with sparsity problem compared to bulk data (Finak *et al.* 2015, Jiang *et al.* 2022). Dropout events are commonly used for defining the observed zero values in single-cell RNA sequencing data, which contain two types of zeros: biological zeros, representing the biologically true loss of expression, and technical zeros, caused by limitations in sequencing technology. The degree of sparsity depends on the technical platform of single-cell RNA sequencing, the depth of sequencing, and the underlying gene expression (Lähnemann *et al.* 2020). This inherent sparsity in scRNA-seq data can pose the huge challenges for downstream analysis, such as clustering and pseudotime analysis.

To address the issue of dropout events in scRNA-seq, various methods have been developed, categorized as model-based imputation (Chen and Zhou 2018, Huang *et al.* 2018, Li and Li 2018, Miao *et al.* 2019, Liu and Li 2023), data smoothing (Gong *et al.* 2018, Van Dijk *et al.* 2018), and methods that reconstruction data through matrix decomposition (Chen *et al.* 2020, Pan *et al.* 2021, Linderman *et al.* 2022) or machine learning (Arisdakessian *et al.* 2019, Gu *et al.* 2022, Shi *et al.* 2023). Data smoothing methods generally adjust all expressed values, whereas model-based imputation methods typically leverage probabilistic models to distinguish technical and biological zeros. In existing methods, model-based approaches assume that gene expression data follow a certain distribution limited in application to partial datasets. Similarly, matrix decomposition in reconstruction-based methods visibly relies on the low-rank assumption of the matrix. With the advancement of technology, datasets are becoming larger, and smoothing-based methods have a time advantage in handling large-scale data. However, smoothing-based methods often require clustering as a preprocessing step, and the accuracy of clustering directly affects the subsequent results, with the possible oversmoothing issues.

**Figure 1.**
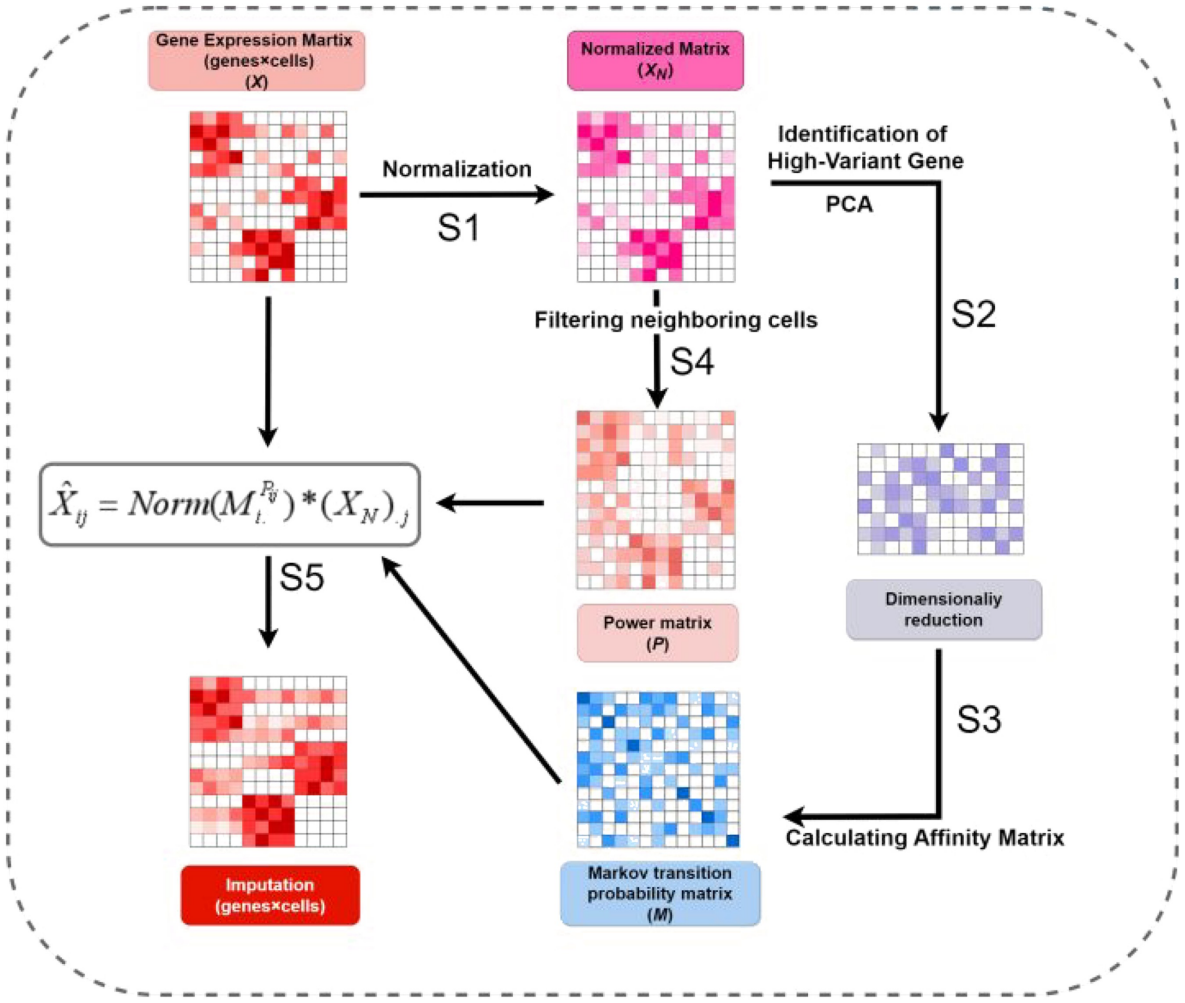
The outline of AcImpute. Step 1: Normalize the matrix. Step 2: Select high-variant genes and proceed with principal component analysis (PCA) dimensionality reduction. Step 3: Apply KNN based strategy to data dimensionally reduced by PCA to obtain neighboring cells and calculate the inter-cellular transition probability matrix. Step 4: Obtain the power matrix by averaging the normalized matrix over its neighboring cells. Step 5: Obtain the final imputation matrix through the computation of formula ${\hat{X}}_{ij}={\mathrm{Norm}(M_{i.}}^{P_{ij}})\;*\;(X_{N}{)}_{.j}$.

In the MAGIC method proposed by Van Dijk *et al.* (2018), the diffusion probability remains constant across all genes in every cell. In contrast, Kharchenko *et al.* (2014) demonstrated the main characteristics of dropout events, the dropout rate could depend on the average expression level of the gene in the population for the given cell. Genes with lower expression levels experience more frequent dropouts. Therefore, if the gene is highly expressed in others cells but exhibits ‘dropout’ in this cell, it could indicate the true expression distinctions among the cells. Inspired by this, we have introduced the AcImpute imputation method. AcImpute can leverage the average expression of similar cells to constrain the diffusion rates of genes with diverse expression levels within cells, thereby preventing over-smoothing. AcImpute can enable highly expressed genes to diffuse more readily among the most similar cells. The specific process is shown in Fig. 1. Finally, we evaluated the performance of AcImpute by comparison with several existing methods using the published datasets.

## 2 Materials and methods

### 2.1 Data preprocessing

Diverse cells may exhibit variations in expression levels during sequencing due to technical factors such as experimental procedures and capture efficiency. Furthermore, varying sequencing depths can also lead to inaccuracies in the estimation of gene expression levels. The input matrix ***X*** requires normalization to obtain matrix $X_{N}$, mitigating the influence of cell size and enhancing comparability of expression values across different cells. The matrix $X_{N}$ is given as

(1) $$(X_{N}{)}_{ij}=\frac{x_{ij}}{\sum_{i=1}^{g}x_{ij}}\;*\;\mathrm{median}\left(\sum_{i=1}^{g}x_{ij}\right),i=1,2,...,g;\;i=1,2,...,n$$

where $x_{ij}$ represents the gene expression value for gene i in cell *j*. After obtaining the normalized matrix $X_{N}$, genes with high variability are selected based on the calculation of the coefficient of variation. The coefficient of variation is calculated by dividing the standard deviation by the mean. Specifically, genes with a mean value ≥0.01 and a coefficient of variation greater than or equal to the first quartile are retained to filter out high-variant genes, resulting in matrix $X_{h}$ for improved clustering in subsequent analyses.

### 2.2 Markov transition matrix

The stable transition probability matrix ***M*** is calculated using matrix $X_{h}$. Firstly, PCA dimensionality reduction of matrix $X_{h}$ can map the data from high-dimensional space to low-dimensional space, thus reducing noise and improving computational efficiency. The affinity matrix ***A*** can be further calculated as

(2) $$A_{ij}={e^{-(\frac{\mathrm{Dist}(i,j)}{\sigma })}}^{2}$$

where Dist(i, j) represents the Euclidean distance between cells, and σ represents the distance between the *k*th nearest neighbor cells of the *i*th cell, whose neighbor cells are obtained through KNN-based strategy. Set the number of neighbors for KNN to a multiple of 3 of *k*, where *k* is equal to 5. The adaptive kernel generates an asymmetric affinity matrix, so it is necessary to use additive method to symmetrize ***A***. Subsequently, the rows of *A* are randomly normalized to obtain the Markov transition matrix ***M***. After raising matrix ***M*** to the power of *t*, a stable transition probability matrix ***M*** is obtained, where the diffusion time *t* is determined by the coefficient of determination (Rseq) of the imputation between *t* and *t* − 1 diffusion.

### 2.3 Calculate the power exponent

Dropout rates vary across different levels of gene expression, with lower expression levels associated with higher dropout rates. This phenomenon is attributed to amplification biases and inherent biological variation (Kharchenko *et al.* 2014). Experimental calculations on six datasets (Zeisel, Baron, Romanov, Chu_cell_type, sc_10x_5cl, and Usoskin) also can confirm that this inverse relationship between gene expression levels and dropout rates. Firstly, the expression values of the same gene across different cell types were summed to obtain the total expression level and the proportion of zero expression values, i.e., the dropout rate, for each gene in different cell types. Then, Pearson correlation coefficients were computed between gene expression levels and gene dropout rates across different cell types for each dataset. The correlation results from different cell types were averaged to obtain the overall correlation result for each dataset. The results for each dataset are as showed in the Table 1. Therefore, it can be observed that gene expression levels and dropout rates are inversely proportional. By normalizing the average gene expression of neighboring cells as the power of the transfer probability, we constrained the transfer probability of genes with differential expression levels between cells. For genes with high expression, dropout data are likely indicative of true zero expression values. Consequently, for genes with higher expression, a lower probability of transfer between cells is anticipated. Specifically, after obtaining the stable Markov transfer matrix ***M***, the first *n* cells with high probability of transition are chosen as the nearest neighbors for each cell, and the transfer matrix is reconstructed using the information from these neighboring cells. For datasets with over 1000 cells, select 100 neighboring cells. For datasets with fewer than 1000 cells, use the following formula to calculate the size of selected neighboring cells:

(3) $$n=\frac{N-15}{1000-15}*100$$

**Table 1.** Correlation between gene expression levels and dropout rates.

Dataset	Correlation
Zeisel	−0.2578
Baron	−0.368
Romanov	−0.261
Chu_cell_type	−0.208
sc_10x_5cl	−0.337
Usoskin	−0.971

Here, 15 refers to the condition that the cell size of the dataset must be >15. After determining the neighboring cell size, the genes from these *n* cells are added based on matrix $X_{h}$ to obtain matrix $X_{a}$. As shown in the following formula:

(4) $${\left(X_{a}\right)}_{i}=\mathrm{mean}(X_{h}(\max_{n}(M_{i})))$$

The range is normalized to 1–3 as the exponent for the stabilized transition matrix, according to the following formula. This involves initial minimum-maximum normalization, followed by scaling the range to 1–3:

(5) $$P=\frac{X_{a}-\min(X_{a})}{\max(X_{a})-\min(X_{a})}*(3-1)+1$$

### 2.4 Imputation of single-cell data

After obtaining the power matrix ***P*** and transition probability matrix ***M***, the imputation matrix $\hat{X}$ is obtained by exponentiating and normalizing each row of the transformation matrix ***M*** and multiplying it with the pre-processed matrix $X_{N}$, following Eq. (6). The higher average expression in similar cells has a larger power, so that the spread among the highly expressed genes is more strongly constrained.

(6) $${\hat{X}}_{ij}=\mathrm{Norm}(M_{i.}^{P_{ij}})*{\left(X_{N}\right)}_{.j}$$

Subsequently, the imputation matrix $\hat{X}$ was rescaled and then subjected to reverse normalization to derive matrix $\bar{X}$, replacing the 0 values in the original matrix with the corresponding values from the matrix $\bar{X}$ to obtain the final result $\bar{X}$.

(7) $$\bar{X}=\{\begin{array}{c} \begin{array}{cc} {\bar{X}}_{ij} & if\begin{array}{cc} X_{ij}=0 & \end{array} \end{array}\\ \begin{array}{cc} X_{ij} & if\begin{array}{cc} X_{ij}\neq 0 & \end{array} \end{array} \end{array}$$

### 2.5 Datasets

To verify the effect of AcImpute, six real datasets (Baron, Zeisel, Romanov, sc_10x_ 5 cl, Chu_cell_type, Usoskin) were used to evaluate the performance of AcImpute and nine other imputation methods in cluster analysis. The six datasets have large spans of cell types and cell numbers to verify the robustness of the method. The input matrix is the count matrix X(g×n), where ‘*g*’ denotes the number of genes, and ‘*n*’ denotes the number of cells. Retain genes with a sum of expression values in the input matrix ***X*** >0.001 and a count of expressed genes in each cell >3. This aims to exclude genes with minimal expression across the majority of cells. After the described preprocessing, the details of the six datasets are summarized in the Table 2. Additionally, the sc_10x_5cl dataset utilized processed results by Hou *et al.* (2020).

**Table 2.** Single-cell datasets for measuring different performance.

Datasets	Number of cell types	Number of cells	Number of genes	Cell source	Dropout rate, %	References
Usoskin	4	622	16301	Clusters of mouse lumbar DRG (dorsal root ganglion)	76.1	Usoskin *et al.* (2015)
Chu_celltype	7	1018	17559	Human embryonic stem cells	45.2	Chu *et al.* (2016)
Baron	14	1937	20125	Human pancreatic islets	86.9	Baron *et al.* (2016)
Romanov	7	2881	18553	*Mus musculus* brain cells	84.0	Romanov *et al.* (2017)
Zeisel	9	3005	18378	Mouse cortex and hippocampus	79.6	Zeisel *et al.* (2015)
sc_10x_5cl	5	3918	10164	Human lung adenocarcinoma cell lines	58.1	Tian *et al.* (2019)

## 3 Experiment

### 3.1 Correlation analysis

**Figure 2.**
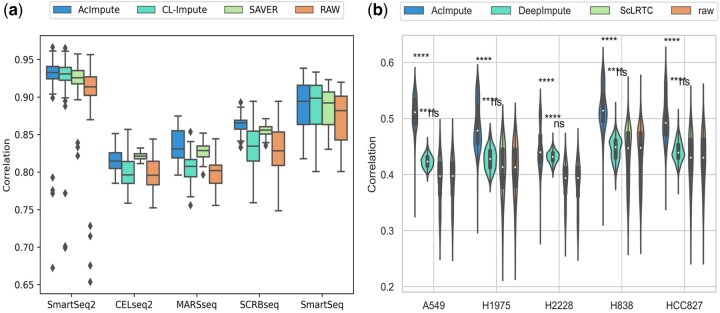
Correlation analysis results. (a) Boxplot results of correlation analysis for the Ziegenhain dataset, (b) correlation analysis results for the sc_10x_5cl dataset.

Two datasets were collected for correlation analysis. These datasets utilized ERCC genes with known concentrations and bulk sequencing data as reference data, respectively. By employing reference data selected from these two different perspectives, the performance of imputation methods in restoring gene expression could be comprehensively evaluated. The first dataset, we selected the Ziegenhain dataset, employed ERCC genes with known concentrations as standards to assess the restoration capability of imputation. Pearson correlation analysis was performed between the imputed and raw data with respect to the reference data, followed by *t*-tests. The correlation analysis results of the Ziegenhain dataset are depicted in Fig. 2a, where ‘Raw’ represents the data before imputation. Across five different sequencing methods (SmartSeq2, CELseq2, MARSseq, SCRBseq, and SmartSeq), AcImpute consistently exhibited a significant improvement relative to CL-Impute, SAVER and the raw data.

As the bulk RNA-seq data are an average expression profile of hundreds of thousands of cells, and ‘dropout events’ are rare, thus the dataset sc_10x_5cl uses bulk RNA-seq data as the reference standard. In this dataset, the experimental replicates of bulk RNA-seq data from the sc_10x_5cl dataset were averaged to serve as the reference standard for calculating correlations, thereby evaluating the restoration capability of imputation. Violin plots and box plots were used to display the spearman correlation coefficients calculated between the imputed data and reference data, as well as between the raw data and reference data. The results distribution of Pearson correlation coefficients for the sc_10x_5cl dataset is illustrated in Fig. 2b, where ‘Raw’ represents raw data, and ‘****’ indicates a *P*-value <0.0001. All five types in the sc_10x_5cl dataset represent cell lines. Despite this finer level of classification, the Pearson correlation results of AcImpute relative to the *t*-test results of DeepImpute, ScLRTC and the raw data are also highly significant. Therefore, AcImpute demonstrates the ability to effectively recover gene expression.

### 3.2 Cluster analysis of real datasets

Unsupervised clustering is a crucial component of downstream analysis in single-cell studies. It is not constrained by prior information, and effective clustering can aid in the discovery of novel cell types, which is of significant importance in fields such as oncology and immunology. Six datasets were collected for clustering analysis: Usoskin, Baron, Zeisel, Romanov, sc_10x_5cl, and Chu_cell_type. The specific details of these datasets are outlined in Table 2. In the clustering analysis, the results of AcImpute were compared with nine different imputation methods: smoothing-based methods (DrImpute and MAGIC), model-based methods (SAVER, scImpute, scRecover, and VIPER), data reconstruction methods based on matrix decomposition (ALRA, ScLRTC, and scRMD), and data reconstruction method based on machine learning (DeepImpute and CL-Impute). After normalizing the data and performing PCA dimensionality reduction and spectral clustering to obtain clustering results, the performance was evaluated using three cluster evaluation indexes: Adjusted Rand Index (ARI), Normalized Mutual Information (NMI), and Purity. The cumulative summation of these indexes was employed to comprehensively assess the quality of clustering results, confirming the effectiveness of AcImpute in clustering analysis.

The total results obtained by summing the three indexes for each of the six datasets were ranked, as shown in Table 3. The results indicate that across the six datasets, ranging in size from 622 cells to 3918 cells, AcImpute consistently demonstrates favorable clustering performance. However, in the Zeisel and Baron datasets, AcImpute did not outperform DrImpute. This discrepancy is attributed to DrImpute integrating results from 10 to 15 clusters, while the Zeisel dataset has 9 clusters and the Baron dataset has 14 clusters. Conversely, in the four datasets with smaller numbers of clusters (4, 7, 7, and 5 clusters, respectively), AcImpute consistently outperforms DrImpute. Additionally, VIPER failed to produce results for the sc_10x_5cl dataset after running for a day, thus its results were not included in the table. Overall, the favorable rankings of AcImpute across these six datasets demonstrate its ability to enhance downstream analysis.

**Table 3.** Ranking of clustering performance for each imputation method across six datasets.

Methods	Usoskin	Chu_cell_type	Baron	Romanov	Zeisel	sc_10x_5cl	Overall Ranking
AcImpute	2	1	2	1	1	2	1
DrImpute	1	2	3	3	2	1	2
ALRA	5	3	4	4	5	3	3
MAGIC	3	13	1	5	4	4	4
SAVER	6	9	9	2	3	7	5
scImpute	4	4	11	8	8	8	7
rawdata	8	10	8	11	7	11	10
scRMD	11	7	7	6	10	12	9
VIPER	10	8	10	11	13	9	11
scRecover	9	12	12	10	11	10	12
DeepImpute	12	11	13	9	12	13	13
ScLRTC	7	6	5	7	6	5	5
CL-Impute	13	5	6	11	9	6	8

**Figure 3.**
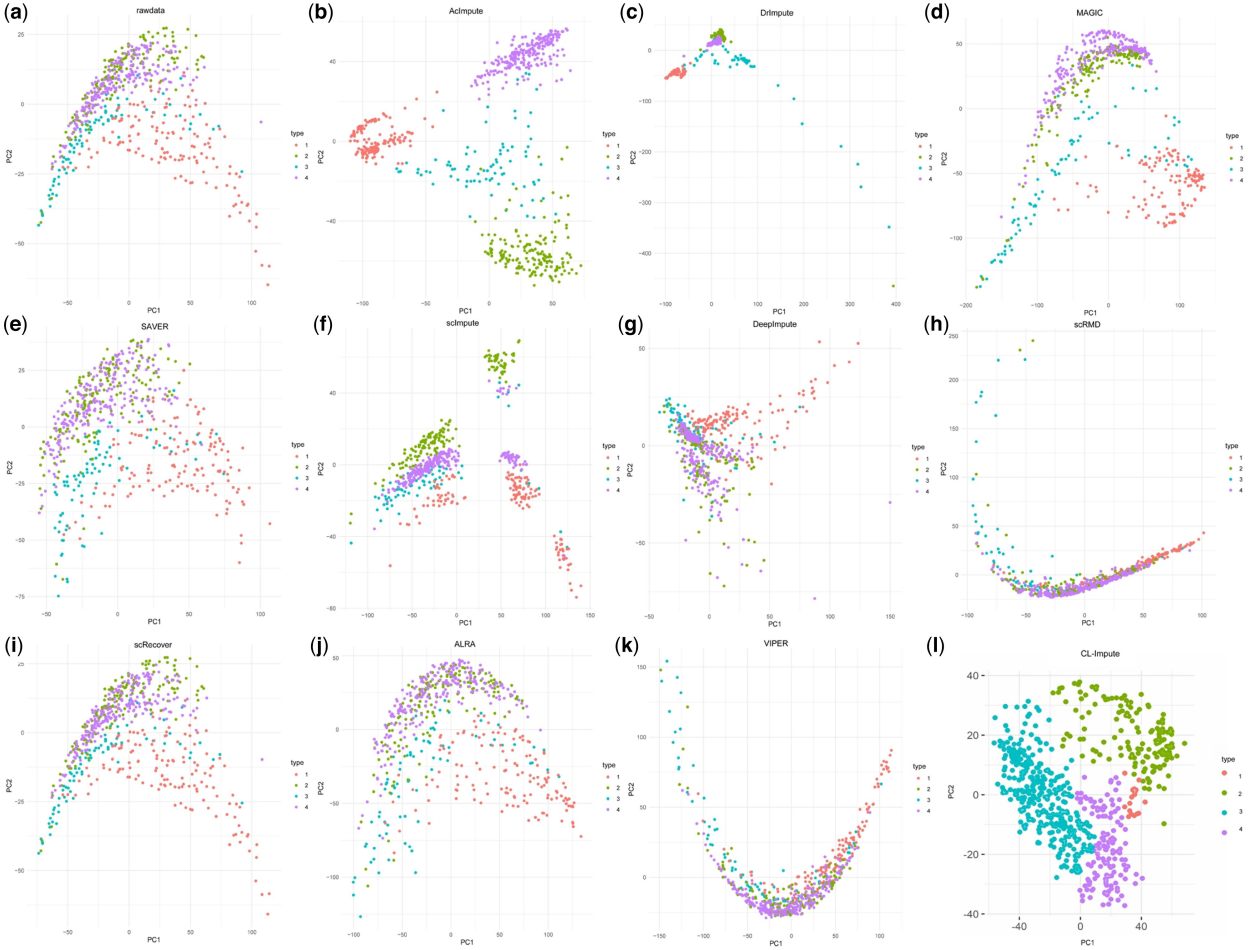
PCA visualization results of raw data and 10 imputation methods on the Usoskin dataset. (a) Rawdata, (b) AcImpute, (c) DrImpute, (d) MAGIC, (e) SAVER, (f) scImpute, (g) DeepImpute, (h) scRMD, (i) scRecover, (j) ALRA, (k) VIPER, and (l) CL-Impute.

To further illustrate the enhancement achieved by AcImpute in clustering, the PCA results of the nine imputation methods on the Usoskin dataset were visualized to demonstrate their ability to identify cell types. Each cell was colored based on its true label. Figure 3 shows the PCA plots of the first two principal components (PCs) for the results of the ten imputation methods on the Usoskin dataset. In the plots, AcImpute can tend to cluster cells of the same type closer while separating cells of different types further apart. Additionally, within the same cell type, individual cells are not densely clustered together. This observation indirectly indicates that AcImpute can alleviate the issue of overly uniform imputation that smoothing-based methods may introduce. Conversely, DrImpute tends to cluster cells very tightly, reflecting the characteristic of over smoothing. Furthermore, there are instances where DrImpute clusters two cell types closely together, as observed in the third and fourth types of the Usoskin dataset in Fig. 3c. This visualization provides a clear demonstration of AcImpute's ability to improve the separation of cell types, AcImpute enhancing clustering performance compared to other imputation methods.

### 3.3 Trajectory inference

Trajectory inference aims to model the gene expression profiles of cells to infer their relative positions during development processes. This method helps reveal cell development trajectories, differentiation pathways, and dynamic changes by describing the temporal changes in cellular states. Monocle2 is a classic trajectory inference algorithm that does not require prior knowledge of gene expression in biological processes, or cell fates in trajectories.

In this section, known temporal states are used as reference labels, and the Monocle2 method is applied to ten imputation data and the raw data of the Chu_time_course dataset for trajectory inference. The Chu_time_course dataset was generated at different time points during the differentiation process in the DEC emergence, specifically at 0, 12, 24, 36, 72, and 96 h.

Firstly, Kendall's tau correlation coefficient and the pseudo-temporal ordering score (POS) metric are selected to quantify the performance of imputation methods in trajectory inference. The basic idea is to measure the consistency between the results and the actual sequence. The results of these two metrics are shown in Table 4. All methods demonstrate improved performance in trajectory inference relative to the raw data. Among them, DrImpute achieves the best results in both POS and Kendall's tau correlation coefficient, with AcImpute ranking second.

**Table 4.** Comparison of POS and Kendall's tau correlation coefficient results for the chu_time_course dataset under 12 imputation methods.

Methods	kendall	POS
DrImpute	0.703	0.876
AcImpute	0.603	0.816
ALRA	0.579	0.746
scImpute	0.514	0.716
DeepImpute	0.376	0.484
SAVER	0.356	0.521
scRMD	0.339	0.496
MAGIC	0.31	0.394
scRecover	0.261	0.332
VIPER	0.249	0.327
CL-Impute	0.433	0.568
ScLRTC	0.227	0.305
Rawdata	0.237	0.322

**Figure 4.**
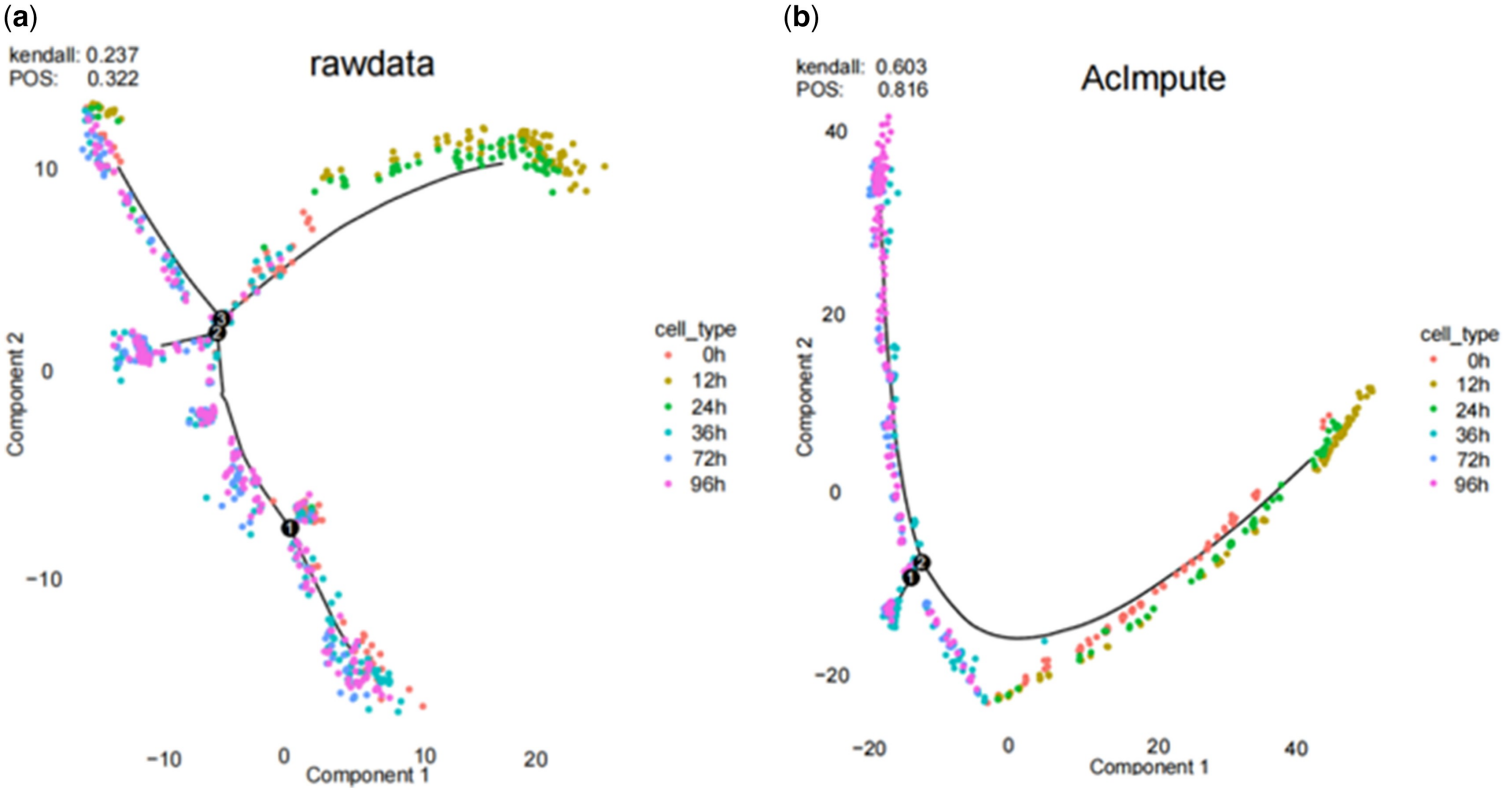
Visualization of cell trajectory inference based on Monocle2. (a) Visualization of trajectory inference for raw data, (b) Visualization of trajectory inference for imputed data using AcImpute.

Furthermore, Fig. 4 depicts the cell differentiation trajectory generated by Monocle2, with cells colored according to their true labels. Data imputation can enhance data quality, and the results indicate that after imputation with AcImpute, cellular dynamic changes are better reflected. In the original data, cells from different time points are relatively dispersed, whereas the results after AcImpute imputation show a clear distinction between 24 and 36 h.

## 4 Results

This paper proposes a smoothing-based imputation method called AcImpute. The innovation of AcImpute lies in reconstructing the transition probability matrix between cells based on the observation that the lower the gene expression level within the same cell type, the higher the dropout rate. Accordingly, AcImpute weights the reconstruction of transition probabilities between neighboring cells more accurately based on the expression levels of genes, thus achieving more precise imputation. When computing the imputation matrix, the weighting of each gene in each cell is different, thereby preserving the diversity among cells and preventing the issue of overly uniform imputation that may arise from smoothing-based imputation algorithms. Experimental results demonstrate that AcImpute effectively alleviates the problem of excessive smoothing.

The experimental section includes correlation analysis, clustering analysis, and trajectory inference. In correlation analysis, datasets with reference data designed from two different perspectives were selected for comprehensive validation. For clustering analysis and visualization, nine methods designed from different perspectives, including model-based, smoothing-based, and reconstruction-based methods, were chosen, and evaluated using three clustering evaluation metrics: ARI, NMI, and Purity. Pseudo-temporal analysis selected POS and Kendall's tau correlation coefficient to evaluate the performance of trajectory inference using the Monocle2 method. The results indicate that AcImpute can effectively recover gene expression and improve the performance of clustering analysis and trajectory inference. Additionally, through the visualization of clustering, it is observed that AcImpute can separate cells of different types, cluster cells of the same type more closely, and maintain a relatively loose distance between cells of the same type, thereby preserving inter-cellular diversity.

However, AcImpute still has room for improvement. The parameter *n*, used in calculating the power exponent matrix, cannot fully adapt. For datasets with many cell types and a small number of cells, choosing 100 nearest neighbor cells when calculating the power exponent matrix may reduce accuracy. Although AcImpute can select fewer nearest neighbor cells for weighting for highly expressed genes, it still cannot completely avoid the situation where biological zeros are filled. In the future, we will continue to address these issues and strive to design a method that can distinguish biological zeros from technical zeros and adaptively select the number of nearest neighbor cells to improve the robustness across different datasets.

Conflict of interest: None declared.

## Data Availability

The dataset Usoskin is available at GEO under accession code GSE59739. The dataset Chu_cell_type and Chu_time_course can available at GEO under accession code GSE75748. The dataset Baron is available at GEO under accession code GSM2230757. The dataset Romanov is available at GEO under accession code GSE74672. The dataset Zeisel is available at http://linnarssonlab.org/cortex/. The dataset sc_10x_5cl uses the processed data obtained from https://github.com/Winnie09/imputationBenchmark. The Ziegenhain dataset is available at GEO under accession code GSE75790.
